# Genetics of Interleukin 1 Receptor-Like 1 in Immune and Inflammatory Diseases

**DOI:** 10.2174/138920210793360907

**Published:** 2010-12

**Authors:** Loubna Akhabir, Andrew Sandford

**Affiliations:** Department of Medicine, University of British Columbia, UBC James Hogg Research Centre, Providence Heart + Lung Institute, Room 166, St. Paul's Hospital, 1081 Burrard Street, Vancouver, BC V6Z 1Y6, Canada

**Keywords:** Asthma, genetics, IL1RL1, immunity, inflammation, respiratory, SNP.

## Abstract

Interleukin 1 receptor-like 1 (IL1RL1) is gaining in recognition due to its involvement in immune/inflammatory disorders. Well-designed animal studies have shown its critical role in experimental allergic inflammation and human *in vitro* studies have consistently demonstrated its up-regulation in several conditions such as asthma and rheumatoid arthritis. The ligand for IL1RL1 is IL33 which emerged as playing an important role in initiating eosinophilic inflammation and activating other immune cells resulting in an allergic phenotype.

An *IL1RL1* single nucleotide polymorphism (SNP) was among the most significant results of a genome-wide scan investigating eosinophil counts; in the same study, this SNP associated with asthma in 10 populations.

The *IL1RL1* gene resides in a region of high linkage disequilibrium containing interleukin 1 receptor genes as well as interleukin 18 receptor and accessory genes. This poses a challenge to researchers interested in deciphering genetic association signals in the region as all of the genes represent interesting candidates for asthma and allergic disease.

The *IL1RL1* gene and its resulting soluble and receptor proteins have emerged as key regulators of the inflammatory process implicated in a large variety of human pathologies We review the function and expression of the *IL1RL1* gene. We also describe the role of IL1RL1 in asthma, allergy, cardiovascular disease, infections, liver disease and kidney disease.

## INTRODUCTION

Interleukin 1 receptor-like 1 (IL1RL1), also called T1, ST2, DER4 and FIT-1, is a member of the interleukin 1 super-family [1] but does not bind interleukin 1 (IL1) [2]. IL1RL1 was an orphan receptor until the description of its ligand, interleukin-33 (IL33) in 2005 [3]. Since then, IL33 binding to IL1RL1 has been associated with a variety of disease states and in particular to inflammatory processes as outlined in recent reviews [4, 5]. In the present review, we will focus mainly on the genetic associations of *IL1RL1* with disease. 

## IL1RL1 GENE AND PROTEINS

**Fig. (1):**
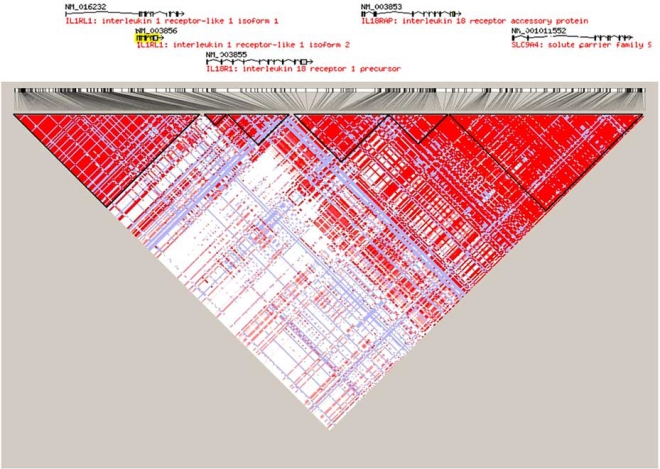
Linkage disequilibrium in IL1RL1 and surrounding genes on chromosome 2q12 (102280 kb to 102500 kb) in the CEU HapMap population.

The *IL1RL1* gene is located in chromosome 2q12 and is composed of 11 exons [6]. A number of IL1 family members reside in the immediate vicinity of the *IL1RL1 *gene namely *IL1R2,* *IL1R1, IL1RL2*, IL18 receptor 1 (*IL18R1)* and IL18 receptor accessory protein* (IL18RAP)*. The region spans about 300 kb and is in high linkage disequilibrium (LD) (Fig. **1**). There is evidence for the involvement of the genes surrounding *IL1RL1* in human and experimental disease, and therefore the causal locus responsible for genetic association signals from this region is difficult to determine.

The *IL18R1 *and *IL18RAP *genes code for the components of the heterodimeric IL18 receptor (the α and β chains, respectively). The cytokine IL18 is a modulator of innate and adaptive immune responses that acts by inducing T helper type 1 (Th1) cell differentiation and T and NK cell maturation or by activating IgE production and T helper type 2 (Th2) cell differentiation under specific cytokine milieus [10-12]. High levels of *IL18* mRNA and protein were observed in lungs of smokers and COPD patients [13] and expression of an alternatively-spliced variant of *IL18R1* was associated with atopy [14]. *IL18R1* expression was also higher in human primary keratinocytes derived from skin lesions of psoriasis and atopic dermatitis patients compared with healthy controls [15]. IL18 signaling has been implicated in host defense [16] and rheumatoid arthritis [17]. Additionally, genetic association data have implicated the IL18 receptor genes in asthmatic and allergic phenotypes [18, 19]. 

### Expression of IL1RL1

The gene transcription is initiated at two separate promoters: a proximal promoter and a distal promoter. The alternative usage of these two promoters leads to differential 3’ processing of the mRNA isoforms [7, 8]. Three known isoforms are produced: isoform 1 which codes for IL1RL1 isoform A (aka ST2L), a long membrane-bound protein, isoform 2 which codes for IL1RL1 isoform B (aka sST2), a short soluble protein and a third isoform which codes for IL1RL1 isoform C (aka vST2) [9], a variant membrane-anchored form of the protein. The soluble form of IL1RL1 corresponds to the extra-cellular domain of IL1RL1 isoform A except for nine amino-acids in the C-terminal region.

IL1RL1 isoform A is mainly expressed on cells of hematopoietic provenance, mainly T cells [20]. It has been shown that binding of IL1RL1 isoform A with its ligand on the surface of basophils, eosinophils and mast cells promotes their activation [21], increased adhesion and survival [22] and degranulation [23], respectively. IL33/IL1RL1 isoform A has also been shown to play a role in activating macrophages [24, 25].

The short form, IL1RL1 isoform B, is expressed by various cells including epithelial cells, endothelial cells, fibroblasts and smooth muscle cells. This expression is augmented upon stimulation with IL1α, IL1β, TNFα, LPS and other factors inducing cell stress such as cardiac infarction and hypoxia [26]. The tissue distribution of IL1RL1 isoform B seems to be relatively ubiquitous, with the highest levels of the secreted form found in the lung followed by the heart and the brain [27].

Several studies show that the membrane-bound IL1RL1 protein acts as a specific marker for Th2 cells [20]. *In vitro* blockade of IL1RL1 signaling with recombinant IL1RL1 protein to compete with the endogenous receptor resulted in the abrogation of differentiation to and activation of Th2, but not Th1, effector cells [28]. Interestingly, IL1RL1 has been found to play a considerable role in a newly discovered immune type2 effector leukocytes, known as nuocytes [29]. An IL13-GFP mouse model was utilized to define these as cells not corresponding to a previously known leukocyte lineage that express ICOS, IL1RL1 and IL25R [29]. The nuocytes’ function included the innate immune response to helminth infection with *Nippostrongylus brasiliensis *by secretion of high levels of IL13 in response to IL25 and IL33.

The ligand for IL1RL1 is a recently discovered member of the interleukin 1 family: IL33 [3]. The signaling of IL1RL1 isoform A binding to IL33 results in the activation of the Mitogen-Activated Protein kinases ERK1, ERK2 and p38 and the subsequent activation of NFκB [3, 23]. IL1RL1 isoform B corresponds to the extra-cellular domain of isoform A and *in vitro* studies have shown that it can also bind IL33 and act as a decoy receptor inhibiting the activation of NFκB [30] and the subsequent inflammatory response. This was confirmed in an animal model where introduction of soluble IL1RL1 decreased pro-inflammatory cytokine (IL4, IL5 and IL13) production in a murine asthma model after treatment with IL33. It was shown that this protective effect of the soluble IL1RL1 seems to be IL10 dependent in an animal model of ischemia reperfusion injury [31].

## IL1RL1 IN DISEASE

### Asthma and other Respiratory Diseases

Increased eosinophil count is a phenotype associated with the majority of asthma cases and correlates with severity of the disease as well as response to glucocorticoid treatment [32]. Using asthma mouse models, it was shown that eosinophilic inflammation is significantly decreased following allergic stimuli in animals subjected to treatments with recombinant IL1RL1 or antibodies directed against the membrane-anchored protein [33, 34]. Soluble IL1RL1 has been shown to be sufficient to reduce experimental allergic airway inflammation using an intravenous IL1RL1 gene transfer mouse model [34], perhaps by acting as a decoy receptor. In addition, a ST2^-/- ^knockdown mouse model of asthma showed decreased airway inflammation [35]. 

IL1RL1 expression has been shown to increase in murine [35] and human [36] asthmatic lungs; soluble IL1RL1 has been shown to increase in the serum of asthmatic patients during acute attacks, and this increase correlated with lung function decrease as well as an increase in the serum levels of the inflammatory cytokine IL5 [37]. Other *in vivo* studies of airway allergic inflammation demonstrated a clear involvement of soluble IL1RL1 protein in regulating a Th2 response after allergen challenge [35] as well as in the resolution of allergen-induced inflammation as assessed by airway hyper-responsiveness [38]. 

Since the late 1990s, genetic studies have shown linkage of chromosome 2 with asthma, lung function (as assessed by FEV_1_%VC, a common clinically-useful index for airflow limitation), eosinophilia and IgE levels [39-41].

Polymorphisms in *IL18R1*, a gene in tight LD with *IL1RL1*, were associated with asthma, atopic asthma and airway hyper-responsiveness using a candidate gene approach in a Danish population and the association consistently replicated in two other European populations [18]. In the same year, another candidate gene association study documented significant genetic association of the gene cluster containing *IL1RL1, IL18R1* and *IL18RAP* with asthma and atopy in a Dutch population [19]. Additional association evidence was reported by the same group using pathway analysis to detect gene-gene interactions in the Toll Like Receptor (TLR)-related pathway. IL1RL1 isoform B has been shown to down-regulate gene expression of TLR4 and TLR1 *in vitro* after treatment with LPS and *in vivo* in a LPS-induced shock mouse model [42].

Twenty-nine genes implicated in TLR regulation were selected for a pathway analysis in Dutch populations [43]. *IL1RL1* SNPs were associated with allergy and asthma phenotypes as single SNPs although the significance did not survive multiple testing correction. In addition, when gene x gene interactions were tested using the multifactor dimensionality reduction approach, *IL1RL1* SNPs were identified as interacting factors in analyses of IgE phenotypes [43].

In a study performed by our group in collaboration with others, we investigated three Canadian and one Australian populations but failed to detect any significant association with *IL1RL1* that survived correction for multiple comparisons [44]. The same cohorts, in addition to one American population, were used in an association study of genes in the vitamin D pathway with asthma and atopy phenotypes. *IL1RL1 *SNPs were selected for this study based on the fact that *IL1RL1* was shown to be transcriptionally regulated by vitamin D [45]. The genotyping covered more variants of *IL1RL1* than the initial study and the number of candidate genes was substantially less (11 versus 120 genes). Significant associations of these variants were observed with asthma and atopy phenotypes [46]. 

Given the role of eosinophils in the pathogenesis of asthma, alleles that associate with increased eosinophil count could be detrimental in terms of asthma risk and severity. In a Genome Wide Association Study (GWAS) of eosinophil count in an Icelandic population, a SNP in *IL1RL1* (rs1420101) showed the most significant association. The A allele of rs1420101 associated with increased eosinophil count and in further analyses with increased serum IgE as well as with three asthma phenotypes (asthma, atopic asthma, non atopic asthma) in nine European populations and one east Asian population [47]. rs1420101 is an intronic SNP which is in high LD (r^2 ^greater than 80%) with a large number of other variants in *IL1RL1*, *IL18R1* and *IL18RAP*; this group of SNPs contains mostly intronic SNPs in addition to a coding-synonymous and a few 3’ and 5’ UTR SNPs. No functional studies have been performed thus far to determine the association-causing SNP.

It is of note that an association of a SNP in *IL33* (rs3939286) with eosinophil count, asthma and atopic asthma was reported in same study, although the *IL33* association with eosinophil count did not reach genome-wide significance. The same *IL33* SNP was associated with nasal polyposis in a Belgian population in a candidate gene study [48].

Wu *et al*. used GWAS data of childhood asthma in a Mexican population [49] to perform a candidate gene analysis. In this study, 237 genes were selected from human and animal model published studies of asthma to have at least one SNP associated with an asthma phenotype. They reported *IL1RL1* among the most significant associations. Furthermore, their results were subjected to multi-marker analysis, which confirmed *IL1RL1* as a significant finding as well as *IL18R1*.

Collectively, there is strong evidence for genetic association of *IL1RL1* with asthma and related phenotypes. This association is certainly very well supported by the biology of IL1RL1 and related proteins. IL33 is secreted by the airway epithelium in response to stress such as allergens or viruses, and binds to IL1RL1 isoform A on the surface of immune cells. There are excellent reviews about the central role of the epithelium in initiating and sustaining immune responses [50]; IL33/ IL1RL1 isoform A plays a crucial role in that process. 

The binding of IL1RL1 isoform A and its ligand IL33 triggers the NFκB signaling pathway, which leads to the transcription of cytokines needed for a Th2 immune response. However, the role of IL1RL1 isoform B remains unclear. Several animal models and *in vitro* studies show that IL1RL1 isoform B prevents the IL33/ IL1RL1 isoform A signaling and consequently attenuates inflammation, indicating its role as a negative regulator of the pro-inflammatory IL33/ IL1RL1 isoform A axis. Human data on the other hand clearly demonstrate a consistent increase of IL1RL1 isoform B in an array of pathological conditions as well as the correlation of this increase with severity. Additionally, there was a report of an animal study showing that mice deficient in IL1RL1 showed attenuated airway inflammation after challenge with an allergen [51], suggesting that IL1RL1 isoform B might be participating in the excessive inflammation observed in asthma. However, the model used for this study was the transgenic TCR-mouse model; these animals are pre-disposed to autoimmune disorders because they carry rearranged TCR α and β genes from a diabetogenic T cell clone.

The above studies do not seem to be consistent with the antagonist role of IL1RL1 isoform B but rather indicate a possible involvement in the pathology. An alternative explanation would be that the increase of IL1RL1 isoform B is a means of preventing an exaggerated immune response but either occurs too late or is insufficient to remedy to the pathological state.

Evidently, soluble IL1RL1 plays a role in the regulation of the immune response, notably in severe disease. Exactly what that role is and the mechanisms underlying it need to be clarified in order to develop efficient strategies for developing therapeutics using the IL1RL1 proteins.

Recent human data in Chronic Obstructive Pulmonary Disease (COPD) seem to indicate an involvement of soluble IL1RL1 in the early stages of COPD [52]. This study however involved a small number of patients and needs replication.

### Allergy and Immune Disorders

A SNP in the distal promoter region of *IL1RL1*, rs6543116 (-26999G/A), was associated with increased risk for atopic dermatitis and up-regulation of gene expression [53]. This study suggested a functional effect of rs6543116 as the A allele correlated with an up-regulation of the gene transcription as well as serum levels. The same group reported the association of serum levels of IL33 and SNPs in the *IL33* gene with Japanese cedar pollinosis, the most common form of allergic rhinitis in Japan [54]. In addition, Castano *et al*. found a protective association of *IL1RL1* SNPs with chronic rhinosinusitis using a cohort of surgery-unresponsive chronic rhinitis patients, this association was stronger in more severe disease [55].

IL33 signaling through IL1RL1 was shown to be involved in anaphylactic shock in an animal model study examining the response of IgE-sensitized mice to IL33 treatment [23]. The same authors had shown elevated IL33 levels in the serum of atopic patients undergoing surgery; this effect was demonstrated to derive purely from innate immunity as T or B cells were not required. The pathological effect could be prevented by treatment with anti-IL33 antibody or soluble IL1RL1 and was not observed in ST2^-/-^ animals [23]. 

*	IL1RL1* and closely linked genes have been implicated in an array of autoimmune diseases. Levels of IL1RL1 isoform B have been shown to be increased in various conditions such as Systemic Lupus Erythematosus (SLE), sclerosis, and rheumatoid arthritis (RA) [56]. Mok *et al*. found that elevated serum IL1RL1 isoform B levels in SLE patients correlated with disease activity [57]. To date, GWAS performed in Chinese and European populations have not found association of *IL1RL1* SNPs with RA [58, 59].

Studies in animal models demonstrated that recombinant IL1RL1 isoform B protein, or anti-IL1RL1 antibody could significantly attenuate the severity of experimental arthritis [60, 61] and IL1RL1 knock-out mice were shown to develop less severe form of disease and had reduced pro-inflammatory cytokine production. Additionally, human studies have shown increased levels of IL33 and IL1RL1 in RA synovium paralleling increased inflammation [62]. Studies in animal models strongly suggest that the involvement of IL33/IL1RL1 in RA is through triggering mast cell degranulation in the RA synovium [63]. Although there is good evidence for a role of IL33/IL1RL1 in human and experimental arthritis, no SNPs in these genes were found associated with susceptibility to RA in GWAS data [64].

The IL1RL1/IL33 signaling axis was implicated in inflammatory bowel disease (IBD) for the first time in two recent studies characterizing IL1RL1 and IL33 protein and mRNA expression in IBD patients [65]. There was an increase in soluble IL1RL1 levels in the gut, which was mainly associated with the active state of ulcerative colitis, indicating a possible negative regulation of the IL1RL1/IL33 pathway in order to dampen the inflammation. Pastorelli *et al*. confirmed the observation of elevated levels of IL1RL1 and IL33 in the serum and mucosa of IBD patients; they also showed that anti-TNF decreased IL1RL1 isoform A levels and increased the soluble isoform making more decoy receptor available in order to sequester IL33 and reduce the inflammation [66].

A SNP 1.5 kb downstream of *IL18RAP* (rs917997) was associated with susceptibility to IBD in a Dutch population; the same SNP was associated with celiac disease in three European populations [67]. rs917997 along with another SNP in the intergenic region between *IL1RL1 *and* IL18R1 *(rs13015714) were associated with celiac disease in a GWAS of a UK population [68]. The same SNP downstream of *IL18RAP *(rs917997) was associated with Crohn's disease in a GWAS [69].

These genetic and mechanistic data suggest that IL1RL1/IL33 plays a role in the gut mucosa similar to the airway epithelium i.e. IL1RL1 isoform A/IL33 eliciting a Th2 immune response and IL1RL1 isoform B serving as a negative regulator.

There is evidence that IL1RL1 directly acts on macrophages to suppress their ability to produce pro-inflammatory cytokines [42]. Macrophages are instrumental in diabetes pathogenesis. In an animal model of diabetes (multiple low-dose streptozotocin-induced diabetes), Mensah-Brown *et al*. [70] showed that specific disruption of the *IL1RL1* gene significantly enhanced inflammation in their mouse model as estimated by an increase in cellular infiltration in pancreatic islets and a reduction in cells immuno-positive for insulin. Recently, a genetic linkage study demonstrated linkage of chromosome 2 with type 2 diabetes with a LOD score of 4.5 [71]. Follow-up genetic studies are warranted to narrow down the linkage signal and investigate specific SNP associations. This may lead to the identification of novel pathways in diabetes.

In summary, the available data on the involvement of IL1RL1 and its ligand IL33 in immune and autoimmune disorders are reasonably consistent; a clearer understanding of the balance between IL1RL1 isoform A/IL33, IL1RL1 isoform B/IL33 and its regulation is needed in order to make that axis a more attractive target for therapeutic intervention.

### Cardiovascular Disease

*In vitro *and animal model studies have demonstrated that IL33/IL1RL1 isoform A signaling protects cardiomyocytes from apoptosis by suppressing Caspase 3 activity and promoting the expression of anti-apoptotic proteins *in vitro* and improves survival in experimental myocardial infarction (MI) animals [72]. Human studies have shown an increase of soluble IL1RL1 after myocardial stress or injury, and MI [26, 73]; the levels correlated with diastolic load [74], cardiac abnormalities on electrocardiogram (ECG) and poor prognosis in dyspneic and MI patients [75-77]. In a study following 150 patients admitted to hospital with acutely destabilized heart failure, multiple serum samples were collected between admission and discharge and soluble IL1RL1 levels were measured. The results showed that IL1RL1 isoform B levels were a powerful predictor of 90-day mortality. Indeed, IL1RL1 isoform B serum levels are considered a reliable biomarker for heart failure [78, 79] as delineated by a recent review by Moore *et al*. [80]. A more recent study demonstrated for the first time that IL1RL1 isoform B could be used to predict left ventricular and infarct recovery after acute MI [81].

The company Critical Diagnostics in collaboration with the Brigham and Women’s Hospital in Boston has developed a diagnosis kit called Presage that uses soluble IL1RL1 levels for diagnosis and prognosis of cardiovascular disease. However, this kit is not yet approved by the FDA for clinical use. Many U.S. and international patents protect the use of IL1RL1 for the diagnosis and prognosis in cardiovascular disease.

The fact that increased level of IL1RL1 is correlated with poor prognosis in different instances of cardiovascular disease points to a role of soluble IL1RL1 as marker for the severity of the immune response. Increased IL1RL1 isoform B is indicative of an overwhelming immune response that is hard to control and thus leads to unfavorable outcome in cardiovascular disease patients, such as after an MI.

### Infections

IL1RL1 isoform B levels correlate with sepsis severity and outcome [82]. A possible mechanism was recently described by Alves-Filho *et al*. [83]. Using the cecal ligation and puncture model in Balb-c mice [83], a widely used model for experimental sepsis, this group demonstrated that IL33 treatment was protective from peritonitis and enhanced bacterial clearance. Their data also show that the protective effect of IL33 treatment was achieved *via *the inhibition of a TLR-signaling-induced protein, GRK2. GRK2 plays a prominent role in sepsis as it down-regulates CXCR2 (a receptor for IL8, a chemokine that attracts neutrophils to infection sites) thus leading to inefficient clearance of bacteria.

In agreement with the role of IL1RL1 proteins in the promotion of Th2 responses, mRNA levels of both receptor and soluble forms of the IL1RL1 transcript were shown to be up-regulated in an animal model of *Toxoplasma gondii* parasitic infection and this up-regulation correlated with protection from the infection [84]. In addition, ST2^-/-^ knockout mice demonstrated increased susceptibility and more severe disease compared to wild type mice as assessed by weight loss, increased parasite transcript levels and typical disease pathology [84]. In 2008, a small study of a Somali population reported an association of a SNP in the 3’UTR of *IL18R1* (rs3213733) with variability in Rubella vaccine-induced humoral immunity [85]. It is interesting that the same SNP was recently shown to be associated with asthma in two different studies, in Mexican and Japanese populations [47, 86]. As LD patterns differ between populations, this suggests a potential functional role of this SNP in regulating gene expression/function. 

Additional evidence for a role of the IL33/IL1RL1 axis in host defense comes from an animal study showing the protective role of IL33 in intestinal infection with nematodes [87]. 

In summary, IL1RL1 confers protection from infection, which is consistent with its involvement in the Th2 immune response. The increase in IL1RL1 signaling skews T cells to Th2 and prevents a parasite-specific Th1 polarized response.

### Liver and Kidney Disorders

In a candidate gene association study of the course of Hepatitis C in a Japanese population, 103 genes including *IL1RL1* and *IL18R1* were investigated [88]. SNPs in both these genes as well as other genes involved in immune responses were significantly associated with serum levels of alanine aminotransferase (ALT). ALT levels are routinely used as a diagnostic test of liver function and elevated levels are an indicator of infections and other disorders. Nevertheless, this group’s data were not corrected for multiple testing and need to be replicated in other populations. 

An over-expression of IL1RL1 and IL33 mRNA in fibrotic liver was reported using mouse and human tissue sections [89].

## CONCLUDING REMARKS

The *IL1RL1* gene and its resulting soluble and receptor proteins have emerged as key regulators of the inflammatory process implicated in a large variety of human pathologies (see summary Table **1**). 

IL1RL1 is important for both innate and adaptive immunity as IL1RL1 isoform A binding with its ligand IL33 leads to polarization of T helper cells into Th2 and also activates and promotes the degranulation of mast cells. The resulting inflammation is down-regulated by the soluble form of IL1RL1; levels of the latter are recognized as biomarkers for the severity of various conditions. Except for the functional analysis of the *IL1RL1* SNP rs6543116 associated with asthma and atopic dermatitis [36, 53], there has been no functional analysis of the disease-associated variants in *IL1RL1*; functional characterization of genetically-associated variants is necessary to determine the causal pathways leading to expression and/or function changes in the proteins.

As shown by numerous animal model studies, targeting the IL1RL1/IL33 axis is potentially a very promising therapeutic avenue for lung, heart and other immune and inflammatory disorders. In order to move the field forward, it will be important to investigate genetic association of the *IL1RL1* region (including the surrounding genes) in different populations with different LD patterns; this will permit a better understanding of the biology behind this region’s involvement in immune and inflammatory disorders and thus facilitate and focus future therapeutic targeting efforts.

## Figures and Tables

**Table 1:** 

Gene	SNP	Chr. Loc.	SNP Location	Disease/Phenotype	pValue	Study	Population	N	Refs.
IL1RL1	rs949963	102769786	5' near-gene	Childhood asthma	0.033	Candidate gene study_follow-up to GWAS	Mexican	492 cases + both parents	Wu *et al. *- J Allergy Clin Immunol 2010
IL1R1	rs3917289	102781911	intronic	Childhood asthma	0.022	Candidate gene study_follow-up to GWAS	Mexican	492 cases + both parents	Wu *et al. *- J Allergy Clin Immunol 2010
IL1RL1	rs11685424	102926981	5' near-gene	Childhood asthma	0.04	Candidate gene study_follow-up to GWAS	Mexican	492 cases + both parents	Wu *et al. *- J Allergy Clin Immunol 2010
IL1RL1	rs11685480	102927086	5' near-gene	Specific IgE egg 1-2 years	0.02	Candidate gene study-gene-gene interaction analysis	Dutch	3062 children (birth cohort)	Reijmerink *et al. *- Allergy 2010
IL1RL1	rs6543116	102927726	promoter	Atopic dermatitis	0.000007	Candidate SNP study	Japanese	452 cases / 636 controls	Shimizu *et al. *- Hum Mol Genet 2005
IL1RL1	rs1420089	102938389	intronic	Asthma	0.033	Candidate gene study	French-Canadian founder population	72 families	Bossé *et al. *- Respir Res 2009
IL1RL1	rs13431828	102954653	5' UTR	Chronic rhinosinusitis	0.008	Candidate gene study	French-Canadian	206 cases / 196 controls	Castano *et al. *- Am J Rhinol Allergy 2009
IL1RL1	rs13431828	102954653	5' UTR	Childhood asthma	0.0002	Candidate gene study_follow-up to GWAS	Mexican	492 cases + both parents	Wu *et al. *- J Allergy Clin Immunol 2010
IL1RL1	rs1041973	102955468	Cod.non.syn (78Ala>Glu)	Atopy	0.046	Candidate gene study	Canadian birth cohort	98 families	Bossé *et al. *- Respir Res 2009
IL1RL1	rs1041973	102955468	Cod.non.syn (78Ala>Glu)	Course of hepatitis C	0.004	Candidate gene study	Japanese	238 cases	Saito *et al. *- Biochem Biophys Res Commun 2004
IL1RL1	rs1041973	102955468	Cod.non.syn (78Ala>Glu)	Childhood asthma	0.00035	Candidate gene study_follow-up to GWAS	Mexican	492 cases + both parents	Wu *et al. *- J Allergy Clin Immunol 2010
IL1RL1	rs1420101	102957716	intronic	Eosinophil count	5.3 x 10^-14^	GWAS	Icelandic	9392 individuals	Gudbjartsson *et al. *- Nat Genet 2009
IL1RL1	rs1420101	102957716	intronic	Asthma	5.5 x 10^-12^	Candidate gene study_follow-up to GWAS	9 European + 1 East Asian populations	7996 cases / 44890 controls	Gudbjartsson *et al. *- Nat Genet 2009
IL1RL1	rs2160203	102960824	3' UTR	Chronic rhinosinusitis	0.03	Candidate gene study	French-Canadian	206 cases / 196 controls	Castano *et al. *- Am J Rhinol Allergy 2009
IL1RL1	rs1946131	102961929	intronic	Asthma / Atopy	0.015 / 0.050	Candidate gene study	French-Canadian founder population	53 / 42 families	Bossé *et al. *- Respir Res 2009
IL1RL1	rs17027006	102965332	intronic	Total IgE	0.02	Candidate gene study-gene-gene interaction analysis	Dutch	3062 children (birth cohort)	Reijmerink *et al. *- Allergy 2010
IL1RL1	rs1921622	102966067	intronic	Specific IgE egg 1-2 years	0.04	Candidate gene study-gene-gene interaction analysis	Dutch	3062 children (birth cohort)	Reijmerink *et al. *- Allergy 2010
IL1RL1	rs1921622	102966067	intronic	BHR / asthma / Total IgE	0.014 / 0.038 / 0.027	Candidate gene study	Dutch	212 / 193 / 276 families	Reijmerink *et al. *- J Allergy Clin Immunol 2008
IL1RL1	rs10208293	102966310	intronic	Chronic rhinosinusitis	0.03	Candidate gene study	French-Canadian	206 cases / 196 controls	Castano *et al. *- Am J Rhinol Allergy 2009
IL1RL1	rs10208293	102966310	intronic	Specific IgE indoor allergens 6-8 years	0.03	Candidate gene study-gene-gene interaction analysis	Dutch	3062 children (birth cohort)	Reijmerink *et al. *- Allergy 2010
IL1RL1	rs1861246	102966783	intronic	BHR / asthma / Total IgE	0.021 / 0.05 / 0.02	Candidate gene study	Dutch	175 / 163 / 230 families	Reijmerink *et al. *- J Allergy Clin Immunol 2008
IL1RL1	rs1861245	102966906	intronic	Asthma	0.032	Candidate gene study	French-Canadian founder population	101 families	Bossé *et al. *- Respir Res 2009
IL1RL1	rs4988957	102968075	cod.syn	Chronic rhinosinusitis	0.03	Candidate gene study	French-Canadian	206 cases / 196 controls	Castano *et al. *- Am J Rhinol Allergy 2009
IL1RL1	rs10204137	102968212	Cod.non.syn	Chronic rhinosinusitis	0.04	Candidate gene study	French-Canadian	206 cases / 196 controls	Castano *et al. *- Am J Rhinol Allergy 2009
IL1RL1	rs10204137	102968212	Cod.non.syn	Childhood asthma	0.013	Candidate gene study_follow-up to GWAS	Mexican	492 cases + both parents	Wu *et al. *- J Allergy Clin Immunol 2010
IL1RL1	rs10192157	102968356	Cod.non.syn	Childhood asthma	0.013	Candidate gene study_follow-up to GWAS	Mexican	492 cases + both parents	Wu *et al. *- J Allergy Clin Immunol 2010
IL1RL1	rs10206753	102968362	Cod.non.syn	Childhood asthma	0.013	Candidate gene study_follow-up to GWAS	Mexican	492 cases + both parents	Wu *et al. *- J Allergy Clin Immunol 2010
IL1RL1 / IL18R1	rs13015714	102971865	intergenic	Coeliac disease	NS	Candidate gene study_follow-up to GWAS	European (Swedish, Norwegian)	325 families	Amundsen *et al. *- Genes Immun 2010
IL1RL1 / IL18R1	rs12999364	102974129	intergenic	BHR / asthma	0.016 / 0.021	Candidate gene study	Dutch	198 / 185 families	Reijmerink *et al. *- J Allergy Clin Immunol 2008
IL18R1	rs2287037 (C-69T)	102979028	5' near-gene	Coal workers' pneumoconiosis	NS	Candidate gene study	French	200 individuals	Nadif *et al. *- Eur Respir J 2006
IL18R1	rs2287037	102979028	5' near-gene	Asthma	0.024	Candidate gene study	European (Danish, British, Norwegian)	736 families	Zhu *et al. *- Eur J Hum Genet 2008
IL18R1	rs1420099	102980543	intronic	Asthma / Atopic asthma	0.00069 / 0.00008	Candidate gene study	European (Danish, British, Norwegian)	736 families	Zhu *et al. *- Eur J Hum Genet 2008
IL18R1	rs1420098	102984279	intronic	Asthma	0.037	Candidate gene study	European (Danish, British, Norwegian)	736 families	Zhu *et al. *- Eur J Hum Genet 2008
IL18R1	rs1362348	102984624	intronic	Asthma / Atopic asthma / BHR	0.0013 / 0.00024 / 0.048	Candidate gene study	European (Danish, British, Norwegian)	736 families	Zhu *et al. *- Eur J Hum Genet 2008
IL18R1	rs1558627	102984684	intronic	BHR / Total IgE	0.049/.028	Candidate gene study	Dutch	180 / 238 families	Reijmerink *et al. *- J Allergy Clin Immunol 2008
IL18R1	rs2058622	102985424	intronic	Atopic asthma	0.045	Candidate gene study	European (Danish, British, Norwegian)	736 families	Zhu *et al. *- Eur J Hum Genet 2008
IL18R1	rs3771170	102985980	intronic	Humoral immunity to Rubella	0.0003	Candidate gene study	Somali	89 individuals	Dhiman *et al. *- Tissue Antigens 2008
IL18R1	rs3771166	102986222	intronic	Childhood asthma	0.011	Candidate gene study_follow-up to GWAS	Mexican	492 cases + both parents	Wu *et al. *- J Allergy Clin Immunol 2010
IL18R1	rs1974675	102986375	intronic	Asthma / Atopic asthma / BHR	0.00005 / 0.00001 / 0.036	Candidate gene study	European (Danish, British, Norwegian)	736 families	Zhu *et al. *- Eur J Hum Genet 2008
IL18R1	rs1465321	102986618	intronic	Humoral immunity to Rubella	0.009	Candidate gene study	Somali	89 individuals	Dhiman *et al. *- Tissue Antigens 2008
IL18R1	rs2270297	102992675	intronic	Humoral immunity to Rubella	0.0002	Candidate gene study	Somali	89 individuals	Dhiman *et al. *- Tissue Antigens 2008
IL18R1	rs2270297	102992675	intronic	BHR	0.048	Candidate gene study	Dutch	185 families	Reijmerink *et al. *- J Allergy Clin Immunol 2008
IL18R1	rs3213733	102997884	intronic	Humoral immunity to Rubella	0.009	Candidate gene study	Somali	89 individuals	Dhiman *et al. *- Tissue Antigens 2008
IL18R1	rs3213733	102997884	intronic	Asthma	0.0035	Candidate gene study	Japanese	288 cases / 1032 controls	Imada *et al. *- BMC Res Notes 2009
IL18R1	rs3213733	102997884	intronic	Childhood asthma	0.0054	Candidate gene study_follow-up to GWAS	Mexican	492 cases + both parents	Wu *et al. *- J Allergy Clin Immunol 2010
IL18R1	rs1035130	103001402	cod.syn	BHR / asthma	0.048 / 0.046	Candidate gene study	Dutch	174 / 159 families	Reijmerink *et al. *- J Allergy Clin Immunol 2008
IL18R1	rs3755274	103002395	intronic	Humoral immunity to Rubella	0.009	Candidate gene study	Somali	89 individuals	Dhiman *et al. *- Tissue Antigens 2008
IL18R1	rs2241117	103003043	intronic	Humoral immunity to Rubella	0.001	Candidate gene study	Somali	89 individuals	Dhiman *et al. *- Tissue Antigens 2008
IL18R1	rs3771161	103003961	intronic	Humoral immunity to Rubella	0.009	Candidate gene study	Somali	89 individuals	Dhiman *et al. *- Tissue Antigens 2008
IL18R1	rs4851004	103009537	intronic	Childhood asthma	0.0079	Candidate gene study_follow-up to GWAS	Mexican	492 cases + both parents	Wu *et al. *- J Allergy Clin Immunol 2010
IL18R1	rs2287033	103011237	intronic	Childhood asthma	0.0063	Candidate gene study_follow-up to GWAS	Mexican	492 cases + both parents	Wu *et al. *- J Allergy Clin Immunol 2010
IL18R1	rs3732127	103013750	3' UTR	Humoral immunity to Rubella	0.009	Candidate gene study	Somali	89 individuals	Dhiman *et al. *- Tissue Antigens 2008
IL18R1	rs1420094	103015687	3' UTR	Childhood asthma	0.0063	Candidate gene study_follow-up to GWAS	Mexican	492 cases + both parents	Wu *et al. *- J Allergy Clin Immunol 2010
IL18R1	rs1420094	103015687	3' UTR	Atopic asthma	0.0063	Candidate gene study	European (Danish, British, Norwegian)	736 families	Zhu *et al. *- Eur J Hum Genet 2008
IL18R1 / IL18RAP	rs1035127	103019919	intergenic	Crohn's disease	1.2 x 10^-4^	GWAS	Caucasian (American, Canadian, Belgian, French, British)	2325 cases / 1809 controls + 1339 families	Barrett *et al. *- Nat Genet 2008
IL18RAP	rs1420106	103035044	5' near-gene	BHR / Total IgE	0.023 / 0.012	Candidate gene study	Dutch	184 / 234 families	Reijmerink *et al. *- J Allergy Clin Immunol 2008
IL18RAP	rs1420100	103037002	intronic	Lumbar disc signal intensity	0.005	Candidate gene study	Finnish	588 individuals	Videman *et al. *- Arthritis Rheum 2009
IL18RAP	rs2272127	103039873	intronic	Schizophrenia+Herpes seropositivity	0.03	Candidate gene study	Caucasian (Amercican)	478 cases / 501 controls	Shirts *et al. *- Am J Med Genet B Neuropsychiatr Genet 2008
IL18RAP	rs917997	103070568	intergenic	Coeliac disease	8.49 x 10^-10^	Candidate gene study_follow-up to GWAS	Northern European	767 cases / 1422 controls	Hunt *et al. *- Nat Genet 2008
IL18RAP	rs917997	103070568	intergenic	Type 1 diabetes	8.03 x 10^-5^	Candidate gene study	Caucasian (Irish, British, American, Romanian, Danish, Norwegian, Finnish)	8064 cases / 9339 controls	Smyth *et al. *- N Engl J Med 2008
IL18RAP	rs917997	103070568	intergenic	Crohn's disease	2.2 x 10^-6^	Candidate gene study	European	1689 cases / 6197 controls	Wang *et al. *- Hum Mol genet 2010
IL18RAP	rs917997	103070568	intergenic	IBD	1.9 x 10^-8^	Candidate gene study	Dutch	1851 cases / 1936 controls	Zhernakova *et al. *- Am J Hum Genet 2008
Chr 2	-	-	-	Type 2 diabetes	LOD=4.53	Genetic linkage study	African-American	580 families	Elbein *et al. *- Diabetes 2009
IL18RAP	6 Tag SNPs	-	-	Cardiovascular disease	NS	Candidate gene study	European	1416 cases / 1772 Controls	Grisoni *et al. *- BMC Med Genet 2009
IL18R1	5 Tag SNPs	-	-	Cardiovascular disease	NS	Candidate gene study	European	1416 cases / 1772 Controls	Grisoni *et al. *- BMC Med Genet 2009
IL1RL1 / IL18R1	Haplo: rs10206753/rs12999364/rs1420099	-	-	BHR	0.006	Candidate gene study	Dutch	179 families	Reijmerink *et al. *- J Allergy Clin Immunol 2008
IL18R1	Haplo:rs1420099/rs1558627/rs2270297	-	-	Asthma	0.002	Candidate gene study	Dutch	180 families	Reijmerink *et al. *- J Allergy Clin Immunol 2008
IL1RL1	Haplo:rs1921622/rs1861246/rs10206753	-	-	BHR / asthma / Total IgE	0.0009 / 0.0008 / 0.007	Candidate gene study	Dutch	192 / 170 / 245 families	Reijmerink *et al. *- J Allergy Clin Immunol 2008
IL18R1 / IL18RAP	22 candidate SNPs	-	-	Cardiovascular mortality	NS	Candidate gene study	German	142 cases / 1142 controls	Tiret *et al. *- Circulation 2005
IL1RL1	multimarker (11)	-	-	Childhood asthma	2.2 x 10^-4^	Candidate gene study_follow-up to GWAS	Mexican	492 cases + both parents	Wu *et al. *- J Allergy Clin Immunol 2010
IL18R1	multimarker (9)	-	-	Childhood asthma	9 x 10^-3^	Candidate gene study_follow-up to GWAS	Mexican	492 cases + both parents	Wu *et al. *- J Allergy Clin Immunol 2010

**Abbreviations**
            IBD Inflammatory bowel disease BHR Bronchial hyper-responsiveness GWAS Genome-wide association study Chr.Loc Chromosal location based on NCBI build 37.1 Chr 2 Chromosome 2 Haplo Haplotype NS Non significant Cod.non.syn Coding non synonymous SNP Cod.syn Coding synonymous SNP
Same SNPs are highlited with the same color; different colors are merely for ease of viewing, and are inconsequential.
